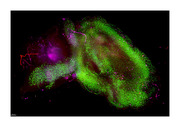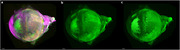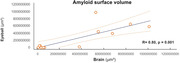# Natural amyloid deposition and neuroinflammation in both brain and eyeball of the old 5xFAD transgenic mouse using Integrated evaluation platform system combining amyloid PET/CT and Macro‐laser light sheet microscopy imaging with tissue clearing

**DOI:** 10.1002/alz.090222

**Published:** 2025-01-09

**Authors:** Hye Joo Son

**Affiliations:** ^1^ Department of Nuclear Medicine, Dankook University College of Medicine, Cheonan, Chungnam Korea, Republic of (South)

## Abstract

**Background:**

Macro laser light‐sheet illuminating microscopy (Macro‐LSFM), allied with tissue clearing technologies, herald a transformative paradigm in biomedical imaging, allowing 3D visualization of neuropathologic networks in a transparent intact mouse brain. Moreover, although traditional focus of AD diagnostic has been on CNS pathology, emerging research points to peripheral amyloid‐beta deposition, specifically in the eyeball, as an avenue for investigation. Coupled with conventional [^18^F]flutemetamol PET/CT imaging, this study leverages the innovative imaging capabilities of Macro‐LSFM with hydrophilic tissue‐clearing technique to elucidate the 3D spatial distribution of AD‐associated neuroinflammation and neuropathologic change both in the brain and eyeball of old transgenic AD mouse.

**Method:**

For thirteen 5xFAD (43 weeks) and seven control mice, we applied a hydrophilic tissue clearing pipelines to extracted brain and eyeball samples, encompassing fixation, clearing, permeabilization, and volume immunostaining using fluorochrome‐conjugated Thioflavin S for β‐amyloid (488 nm), anti‐CD11b for microglia (561 nm), and anti‐ACSA‐2 for astrocytes (647 nm). Macro‐LSFM imaging was performed using a Lightsheet Z.1 microscope (Objective 5x NA 0.16, Zoom 0.75x, single‐mode illumination). 3D volumetric surface models for amyloid, astrocytes, and microglia mapping were generated using Imaris v9.6 software with a minimum surface grain size of 4.0 μm.

**Result:**

In Macro‐LSFM, AD showed significantly higher total surface volumes of amyloid accumulation than control (AD, 898634368 µm³ [383355488‐1324986752]; Control, 33320178 µm³ [11156785‐65390988], p=0.0006). Eyeballs of AD also showed higher amyloid deposits than control (AD, 51091002 µm³ [1002006‐97460784]; Control, 229293 µm³ [8466‐3501462], p=0.0066), showing strong positive correlation of amyloid accumulation between brain and eyeball (R=0.8, p=0.001). AD showed higher anti‐ACSA‐2 specific astrocyte (AD, 59064360 µm³ [27815500‐222619280]; Control 20272722 µm³ [9317288‐27223352], p=0.0047) and anti‐CD11b specific microglial expression (AD, 51210100 µm³ [15309118‐135532144]; Control, 23461593 µm³ [14499170‐27924110], p=0.0162) than control.

**Conclusion:**

Our synergistic amyloid PET/LSFM approach revealed that active neuroinflammation persists in old AD brain, marked by substantial amyloid deposition, challenging the traditional sequence of early neuroinflammation leading to late‐stage neurodegeneration. Our novel observation of correlated ocular amyloid deposition introduces the eye as a promising, accessible site for the early diagnosis of AD pathology.